# Tomato *R*-gene-mediated resistance against Fusarium wilt originates in roots and extends to shoots via xylem to limit pathogen colonization

**DOI:** 10.3389/fpls.2024.1384431

**Published:** 2024-05-01

**Authors:** Margarita Šimkovicová, Gertjan Kramer, Martijn Rep, Frank L. W. Takken

**Affiliations:** ^1^Molecular Plant Pathology, Faculty of Science, Swammerdam Institute for Life Sciences, University of Amsterdam, Amsterdam, Netherlands; ^2^Laboratory for Mass Spectrometry of Biomolecules, Faculty of Science, Swammerdam Institute for Life Sciences, University of Amsterdam, Amsterdam, Netherlands

**Keywords:** wilt disease, tomato grafting, R-genes, proteomics, effectors, pathogenesis-related proteins, plant-fungal interaction

## Abstract

Vascular wilt disease, caused by the soil-borne fungus *Fusarium oxysporum* (*Fo*), poses a threat to many crop species. Four different tomato resistance (*R*) genes (*I-1*, *I-2*, *I-3*, and *I-7*) have been identified to confer protection against *Fo* f.sp. *lycopersici* (*Fol*). These *I* genes are root-expressed and mount an immune response upon perception of the invading fungus. Despite immune activation, *Fol* is still able to colonize the xylem vessels of resistant tomato lines. Yet, the fungus remains localized in the vessels and does not colonize adjacent tissues or cause disease. The molecular mechanism constraining *Fol* in the vascular system of the stem remains unclear. We here demonstrate that an *I-2*-resistant rootstock protects a susceptible scion from Fusarium wilt, notwithstanding fungal colonization of the susceptible scion. Proteomic analyses revealed the presence of fungal effectors in the xylem sap of infected plants, showing that the lack of fungal pathogenicity is not due to its inability to express its virulence genes. To identify mobile root-derived proteins, potentially involved in controlling fungal proliferation, comparative xylem sap proteomics was performed. We identified distinct pathogenesis-related (PR) protein profiles in xylem sap from *Fol*-inoculated *I-1*, *I-2*, *I-3*, and *I-7* resistant lines. Despite structural diversity, all four immune receptors trigger the accumulation of a common set of four PR proteins: PR-5x, PR-P2, and two glucan endo-1,3-β-D-glucosidases. This research provides insights into Fusarium resistance mechanisms and identifies a core set of proteins whose abundance correlates with defense against Fusarium wilt.

## Introduction

Vascular wilt diseases, caused by bacteria, fungi, or oomycetes, impact a wide array of economically important crops globally (Oliver et al., 2009; Planas-Marquès et al., 2020; Chitwood-Brown et al., 2021). Fusarium wilt is caused by *Fusarium oxysporum*, a soil-borne fungal pathogen that penetrates root tissue and colonizes xylem vessels, resulting in leaf epinasty, wilting, and eventually plant collapse (Gordon, 2017). Presently, the most effective approach to control Fusarium wilt in crops involves utilizing Resistance (*R*) genes. R-proteins, upon detection of a corresponding avirulence (Avr) effector, initiate an immune response termed effector-triggered immunity (ETI) that prevents disease development (Takken and Joosten, 2000).

The interaction between tomato (*Solanum lycopersicum*) and *Fusarium oxysporum* f.sp*. lycopersici* (*Fol*) serves as a well-established pathosystem to investigate the molecular basis of disease and resistance to vascular pathogens (Gordon, 2017). Within this system, four distinct *R*-genes have been identified and cloned that confer *Fol* resistance. Three of them are employed in commercially cultivated tomato varieties; *I-1, I-2*, and *I-3* (Takken and Rep, 2010; Chitwood-Brown et al., 2021). These genes, known as “Immunity” or I genes, encode structurally diverse immune receptors recognizing matching Avr proteins from the fungus. I-3 (an S-locus receptor-like kinase), I-1, and I-7 (Leucine-rich repeat (LRR) - receptor-like proteins) are membrane-localized receptors recognizing the presence of Avrs in the extracellular space (Catanzariti et al., 2015, 2017; Gonzalez-Cendales et al., 2016). In contrast, the I-2, (Nucleotide-binding – LRR protein) receptor functions intracellularly (Simons et al., 1998; Houterman et al., 2009) recognizing Avr2 in the nucleus (Ma et al., 2013). Interestingly, the expression patterns of these genes differ; besides expression in tissues not infested by *Fol*, *I-2* and *I-7* are expressed in both the roots and stems, whereas *I-1* and *I-3* are only expressed in roots (Kajala et al., 2021). Tomato plants carrying *I-1* exhibit resistance to *Fol* race 1, *I-2* to race 2, and either *I-3* or *I-7* to race 3. I-1, I-2, and I-3 recognize the corresponding Avr1, Avr2, and Avr3 proteins, respectively (Rep et al., 2004; Houterman et al., 2008, 2009). The Avr recognized by I-7 remains unknown (Gonzalez-Cendales et al., 2016). The commonality between the structurally diverse and differentially expressed I receptors is that they all confer resistance to the same fungus, but it is as yet unknown whether the mechanisms restricting the pathogen differ.

ETI typically fully blocks the spread of a pathogen in a host, as exemplified by e.g. tomato *Cf-9* containing the fungus at the infection site in the leaf (De Wit et al., 1985). Vascular pathogens, however, are rarely fully restricted by *R* genes and colonize the vasculature of resistant plants to various extents. Besides *Fol, Verticillium dahliae*, *Ralstonia solanacearum,* and *Xanthomonas oryzae pv. oryzae* have been shown to proliferate within the vasculature of resistant hosts (Yoshimura et al., 1998; van der Does et al., 2019; Planas-Marquès et al., 2020; Liu et al., 2023). The degree of *Fol* colonization in a resistant line depends on the *R*-gene present, indicating quantitative distinctions in the immune responses. Differences among resistant lines are specifically noticeable in the extent of plant stem colonization, rather than in the colonization pattern of the root epidermis or cortex (van der Does et al., 2019). Whereas colonization of the stem vasculature occurs in *I-2* tomato plants, the fungus is contained in the xylem vessels and does not escape from the vasculature to colonize adjacent tissues (Mes et al., 2000).

*I-1* rootstocks protect the scion of a grafted susceptible variety against Fusarium wilt, implying that Fusarium resistance is root-based (Heinze and Andrus, 1945). There are contrasting views on how resistant plants constrain *Fol* pathogenicity. Some studies suggest the presence of antifungal compounds in the xylem sap, while others argue for the existence of a physical barrier in the root tissues (Gottlieb, 1943; Snyder et al., 1946). The root-based physical barrier hypothesis was challenged by showing that the introduction of Fusarium conidia in stem cuttings of resistant tomato plants did not result in disease symptoms (Scheffer and Walker, 1954). Subsequently, *in vitro* assays demonstrated direct antifungal activity of xylem sap collected from *Fol* race 1 inoculated resistant tomato plants, lending support to the former hypothesis (Stromberg, 1977).

Based on the expression pattern of the *I*-genes in root tissues and the grafting experiments, resistance to *Fol* appears to be primarily root-based (Heinze and Andrus, 1945; Kajala et al., 2021). Yet, it is unclear what mechanism restricts the fungus in the xylem vessel of the stem and prevents colonization of vasculature adjacent tissues. *I-2* and *I-7* are expressed in stem tissue and might be controlling the fungus in this tissue, however, no detectable expression of *I-1* and *I-3* has been observed in stem tissue of tomato (Kajala et al., 2021). To test whether resistance-controlling compounds are transferred from roots to the shoots, grafting experiments were performed in this study. Susceptible scions were grafted on an *I-2* rootstock and colonization of the shoot was determined. To identify host proteins whose abundance correlates with resistance, and to investigate whether the four *I* genes (*I-1*, *I-2*, *I-3*, and *I-7*) induce accumulation of the same or different proteins, proteomic analysis of xylem sap was performed. To capture early immune responses, xylem sap was collected at one-week post inoculation (wpi). This is an earlier timepoint than that used in our previous studies in which a single xylem-specific Pathogenesis-Related protein 5 (PR-5x) was found to highly accumulate in the xylem sap of *I-2* resistant tomato following inoculation with *Fol.* No fungal effector proteins were detected in the sap (de Lamo et al., 2018). To examine whether the host immune responses compromise the ability of the fungus to produce and secrete effector proteins, the *Fol* effectorome in the xylem sap of the resistant lines was analyzed.

## Materials and methods

### Plant and fungal material

Six different tomato (*Solanum lycopersicum*) genotypes were used in this study. Four of them are wild-type cultivars: C32 (susceptible), KG52201 (susceptible), 90E402F (*I-1*), and E779 (*I-3*) (Kroon and Elgersma, 1993; Simons et al., 1998; Mes et al., 1999; Scott and Jones, 2004), three are transgenic lines; KG324 and KG325 are *I-2*-containing derivatives of cv. KG52201, and MM+*I-7* is a derivative of cv. Moneymaker (Simons et al., 1998; Gonzalez-Cendales et al., 2016). All plants were cultivated in a controlled greenhouse environment, maintained at a temperature of 25°C, 65% relative humidity, and a 16-hour photoperiod. The fungal isolates *Fol004* (race 1), *Fol007* (race 2), and *Fol029* (race 3) were used (Rep et al., 2005).

### Tomato seedling grafting

Sixteen-day-old tomato seedlings were grafted at the hypocotyl using Grafting Cassettes™ (GRA&GREEN Inc, Japan; https://www.gragreen.com/en). The seedlings remained in the grafting cassettes for three weeks inside a propagator to sustain high humidity permitting the formation of graft junctions. The roots of the grafted plants were covered with soil to stimulate root growth. During the first week, the propagators were covered with paper tissue to minimize evaporation. In the third week, propagator humidity was gradually reduced from 90% to 65% to match greenhouse conditions (Aalders et al., 2024).

### Fusarium inoculation assay

Fungal isolates were inoculated from glycerol stocks and cultivated on Czapek’s Dox Agar (CDA) plates at 25°C for a minimum of seven days. From the plate, a single plug was transferred to a 250ml flask containing 100 ml of minimal nitrate medium (100mM KNO3, 3% sucrose, and 0,17% Yeast Nitrogen Base without amino acids or ammonia) and incubated for five days at 25°C, 150 rpm. Fungal spores were filtered through sterile miracloth (22-25μm pore size), pelleted for 10 min at 2000 rpm, and washed with sterile Milli-Q (MQ). Before inoculation tomato roots were washed with water. The concentration of the spore suspension, the extent of root trimming, and inoculation time depended on the age of the plant and the type of experiment. For the fungal recovery assay, the roots of ten-day-old seedlings were trimmed to ~1 cm length and inoculated with 10^7^ spores/ml suspension for two minutes. Untrimmed tomato roots of three-week-old grafted tomato plants were inoculated for five minutes with a 0.5x10^7^ spores/ml suspension. For xylem sap proteomics, the main root and lateral roots of four-week-old tomato plants were trimmed to ~1 cm and inoculated for five minutes with 0.5x10^7^ spores/ml suspension.

Three weeks post-inoculation (wpi) of grafted tomato plants, disease index (DI), and fresh weight (FW) were determined. Disease index was scored on a scale of 0-5 (0, no brown vascular bundles; 1, only browning of vascular bundles at the crown level; 2, less than half of the bundles are brown at cotyledon level; 3, at least half of the bundles are brown; 4, 3/4 of the bundles are brown; 5, dead plants) (de Lamo et al., 2018).

### Fungal recovery assay

For the fungal recovery assay from grafted plants, stems were cut at the crown level and above the first true leaf node at three wpi. The cut stems were surface-sterilized with 70% ethanol for four minutes and rinsed with sterile MQ (de Lamo et al., 2020). From the sterilized stems, slices (~0,5 cm thick) were collected from the first true leaf node, cotyledon, and right below and above the grafting point. For the fungal recovery assay from MM+*I-7* plants, the stems of 19-day-old tomato MM+*I-7* seedlings were submerged in ethanol for 5 seconds, rinsed with sterile MQ, and cut into ~8 mm long fragments (van der Does et al., 2019). Both stem slices and stem fragments were placed on potato dextrose agar plates supplemented with penicillin (100μg/ml) and streptomycin (200μg/ml) and incubated for three to five days at 25°C. For quantifying colonization of stem slices, the following numbering was used; 1= fungal outgrowth from a stem slice right below the grafting point, 2 = right above the grafting point, 3= fungal outgrowth at the level of the cotyledon, and 4= first true leaf node. The surface area of fungal outgrowth from stem slices was quantified using Fiji (https://imagej.net/Fiji) plugin tool.

### Xylem sap collection and sample preparation for LC-MS

Xylem sap was collected one-week post inoculation of four-week-old tomato plants. Plants were generously watered eighteen and three hours before xylem sap collection. The plants were cut just below the second true leaf node, positioned horizontally, and attached to a polystyrene tube placed on ice. The stump exuded xylem sap for four to six hours. Five repetitions of *Fol*-inoculation and xylem sap collection were performed during subsequent weeks. Xylem sap from four to six plants per repetition was pooled and centrifuged at 800 x g for 10 minutes to eliminate potential spores and soil particles. Next, 12 ml of the pooled xylem sap was concentrated using Amicon Ultra-15 Centrifugal Filter Units (Millipore) at 2500 x g for 20-25 minutes. The concentrated samples were transferred to low-binding tubes (Protein LoBind microcentrifuge tubes, Eppendorf). Protein quantification was performed using a BCA kit (BCA1-1KT Sigma Aldrich). Samples were treated with TCEP and CAA at final concentrations of 10 mM and 30 mM, respectively, and incubated at 70°C for 30 minutes for a 1 step reduction and alkylation of disulfide bridges. Subsequently, samples were prepared for mass spectrometry analysis using the single-pot, solid-phase-enhanced sample preparation (SP^3^) protocol (Hughes et al., 2014), with modifications to optimize protein recovery for xylem sap proteins. In short, no detergents were added to the samples to enable optimal precipitation of the soluble xylem sap proteins, and the precipitation time was extended to 30 minutes at room temperature. Following two washes with 70% ethanol and a single wash using acetonitrile, beads were air-dried and resuspended in 100 mM ammonium bicarbonate (Sigma) after which trypsin was added at a protease-to-protein ratio of 1:50 (w/w) at 37°C. Following overnight digestion formic acid was added to achieve a final concentration of 1%, resulting in an approximate pH of 2. The samples were then placed on a magnetic separator device and the peptides were recovered for LC-MS analysis.

### LC-MS and label-free quantification of the proteome

Resulting samples were separated by reversed phase chromatography using an Ultimate 3000 RSLCnano UHPLC system (Thermo Scientific, Germeringen, Germany). Peptide separation was performed on a 75 μm × 250 mm analytical column (C18, 1.6 μm particle size, Aurora, Ionopticks, Australia), which was maintained at a temperature of 50°C and operated at a flow rate of 400 nL/min with 3% solvent B for 3 minutes (solvent A: 0.1% formic acid in water, solvent B: 0.1% formic acid in acetonitrile, ULCMS-grade, Biosolve). Following this, a multi-stage gradient was applied, with 17% solvent B at 21 minutes, 25% solvent B at 29 minutes, 34% solvent B at 32 minutes, 99% solvent B at 33 minutes, kept at 99% solvent B till 40 minutes. The system was returned to initial conditions at 40.1 minutes and was held until 58 minutes for equilibration. The eluted peptides were electrosprayed by a captive spray source via the column-associated emitter and were analyzed by a TIMS-TOF Pro mass spectrometer (Bruker, Bremen, Germany). The instrument was operated in PASEF mode for standard proteomics acquisition, with quadrupole isolation widths set at 2 Th at 700 m/z and 3 Th at 800 m/z, and collision energy values varying between 20 and 59 eV across the TIMS scan range. Precursor ions possessing an m/z range from 100 to 1700 and a TIMS range from 0.6 to 1.6 Vs/cm^2 were chosen for fragmentation. PASEF MS/MS scans were initiated ten times with a total cycle time of 1.16 seconds, a target intensity of 2e4, an intensity threshold of 2.5e3, and a charge state range of 0-5. Active exclusion was enabled for a period of 0.4 minutes, with precursors being reevaluated if the ratio of the current intensity to the previous intensity exceeded 4.

LC-MS data were processed using MaxQuant software (version 1.6.10.43) using standard settings, i.e. trypsin/p as the enzyme allowing for 2 missed cleavages with carbamidomethylation at cysteine as a fixed modification and oxidation at methionine as a variable modification searching the proteome databases of *Solanum lycopersicum* (UP000004994) and *Fol* race 2 (UP000009097) from Uniprot (03-2022). Several discrepancies exist in the current tomato Ensembl genome, wherein a multi-exon gene has been erroneously assigned for the single-exon *PR5x* gene (gene ID: *Solyc08g080620*; protein ID: Q8LPU1) and NP24 (gene ID: *Solyc08g080640*; protein ID: P12670). Additionally, the N-terminal sequence of P23 (gene ID: *Solyc08g080650*; protein ID: Q01591) is lacking nine amino acids. In the interim, we manually included Q8LPU1, modified Q01591, and deleted the incorrect A0A3Q7HTH3 and A0A3Q7HVV0 entry. The fungal databases were improved by adding non-annotated sequences of *Fol* Avr proteins. Bioinformatics analyses of MaxQuant output were performed with Perseus 2.03. Principle component analyses labeled one mock- and one *Fol*-inoculated *I-1* plant repetition as outliers, which were excluded from further investigations. Before statistical analysis contaminants, reversed hits, and proteins identified in only one of the five repetitions were excluded. Subsequently LFQ values were log2 transformed and missing values were imputed using the lowest value in the dataset (-9.32). Differential accumulation of proteins between *Fol*- and mock-inoculated plants was determined via a Student’s T-test (p-value) without restricting the p-value correction using FDR or Benjamini Hochberg analysis.

## Results

### An *I-2* resistant rootstock prevents *Fol* disease development in a susceptible scion, notwithstanding the presence of the fungus in the scion

**Figure 1:**
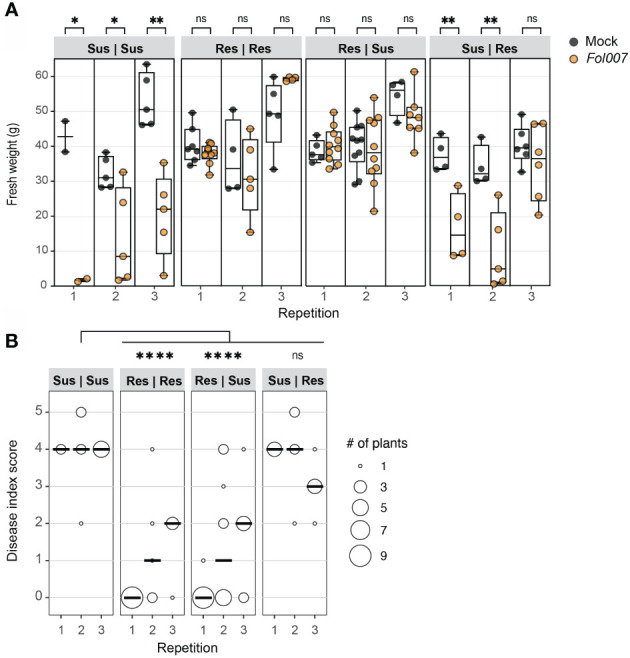
Resistant (*I-2*) tomato rootstocks protect susceptible scions from Fusarium wilt disease. *Fol007* (race 2) resistant (Res) and susceptible (Sus) plants were grafted in four different combinations (rootstock | scion). Approximately five-week-old chimeric plants were either mock- or *Fol00*7-inoculated. Disease scoring of the grafted genotypes occurred three wpi. **(A)** Tukey boxplot shows plant fresh weight above the cotyledon level of three independent repetitions. Each data point is represented by a filled black circle (Mock) or an open black circle (*Fol*). Significant differences between mock- and *Fol*-inoculated plants were determined using Student’s unpaired t-test (*p ≤ 0.05; **p ≤ 0.01) ns = non-significant. **(B)** The dot plot shows the disease index of grafted genotypes. The scores from three repetitions were merged and tested using a Mann-Whitney U statistical test (*p ≤ 0.05; ****p ≤<0.0001).

*Fol* induces wilt disease in susceptible tomato plants by extensively colonizing xylem vessels and subsequently spreading to the adjacent tissues in the root and stem (Gordon, 2017). In an incompatible interaction, *Fol* also colonizes above-ground tissue but remains confined within the vessels, without causing visible disease symptoms (Mes et al., 2000). To investigate the potential of a resistant rootstock in preventing *Fol*-induced disease and colonization of susceptible foliage, *Fol* disease assays were conducted using chimeric plants composed of *Fol007* (race 2)-susceptible (Sus) (KG52201) and *I-2* resistant (Res) tissue (KG325). It was hypothesized that resistance is xylem-based, allowing a resistant rootstock to confer resistance to a susceptible scion, despite the presence of the fungus in a susceptible scion. Four grafting combinations were employed (rootstock | scion): Sus | Sus, Res | Res, Res | Sus, and Sus | Res. Plants were grafted at the hypocotyl and three weeks after mock- or *Fol007*-inoculation, fresh weight and disease index at the cotyledon level were assessed. All mock-inoculated graft combinations per repetition exhibited similar fresh weights showing that the tissues are compatible (**Figure 1A**). As expected, *Fol007*-inoculated Sus | Sus grafts displayed a significant decrease in fresh weight in all three repetitions, with most of the chimeric plants showing severe vascular browning (DI=4) (**Figures 1A, B**). Similar results were obtained for Sus | Res grafts in the first and second repetition, showing that a susceptible rootstock results in severe disease symptom development in the resistant scion. In the third repetition, *Fol*-inoculated Sus | Res grafts did not display a significant decrease in fresh weight but showed clear vascular browning (DI=3) at the cotyledon level (resistant tissue). Conversely, the fresh weight of both *Fol007*-inoculated Res | Res and Res | Sus grafts resembled those of mock-inoculated grafts. The disease indexes of Res | Res and Res | Sus results were very similar to each other, with the majority exerting none or minor vascular browning (DI=0-2). Merged disease index scores show significant differences between Sus | Sus and Res | Res or Res | Sus, while no significance was found between Sus | Sus and Sus | Res. These observations demonstrate that a resistant rootstock prevents disease development in a susceptible scion.

**Figure 2:**
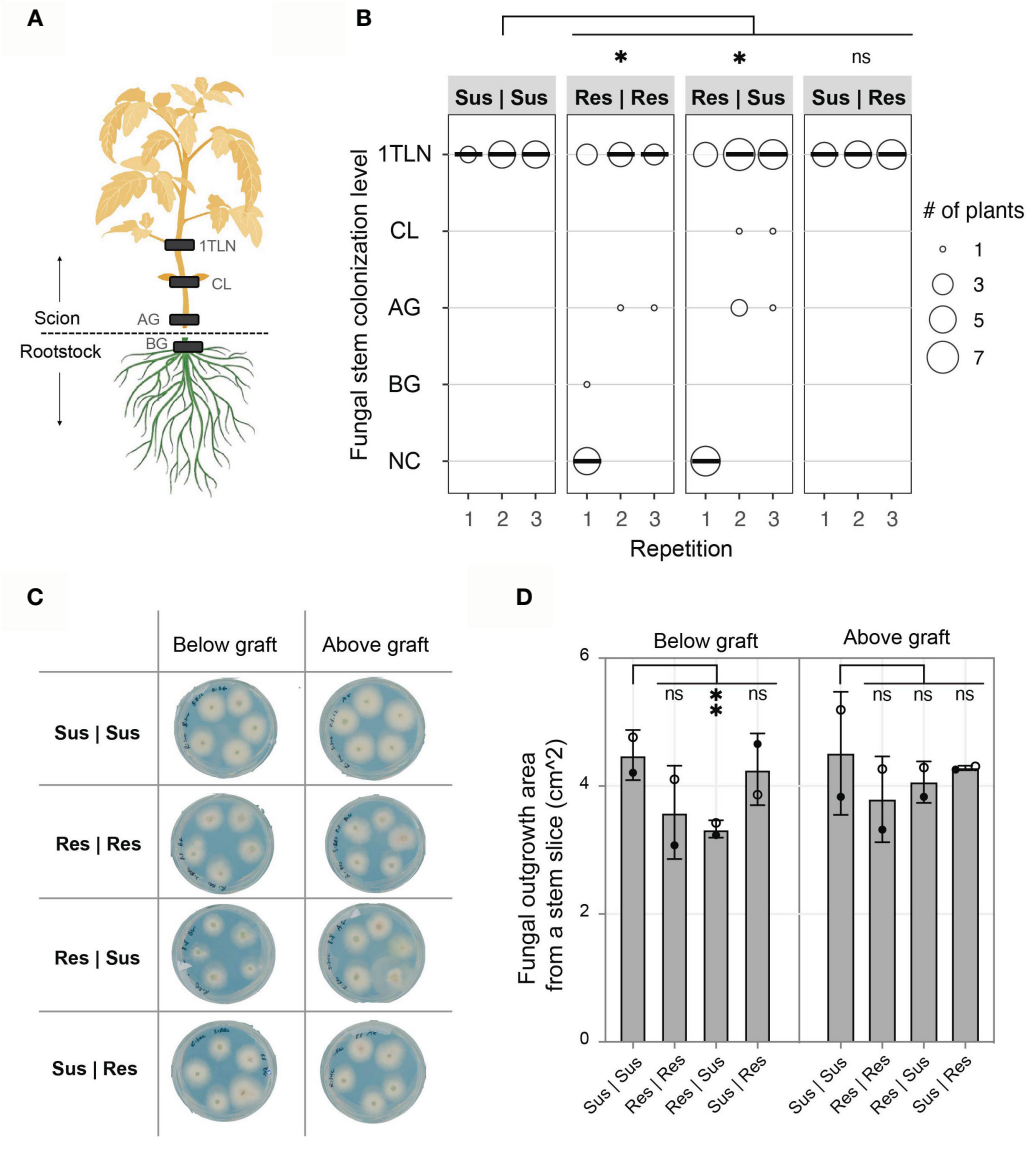
*I-2* resistant (Res) tomato rootstocks reduce colonization of *Fol* in susceptible (Sus) scions. **(A)** Stem sections from below the graft (BG), above the graft (AG), cotyledon (CL), and 1^st^ true leaf node (1TLN) were monitored for the presence of *Fol007* (race 2). The absence of *Fol* from each stem level was described as “no colonization” (NC). **(B)** The dot plot shows fungal stem colonization scores of different grafting combinations (rootstockscion). The scores from three repetitions were merged and tested using a Mann-Whitney U statistical test (*p ≤ 0.05). **(C)** Representative scan of plates from the 2^nd^ repetition showing *Fol* outgrowth from BG and AG stem slices. Each plate includes five stem slices from individual plants. **(D)** The column chart shows the surface area (cm^2) of *Fol* outgrowth from BG and AG stem slices from two repetitions. Open black circles (2^nd^ repetition) and filled black circles (3^rd^ repetition) represent the average of four to eight stem slices per grafting combination. The scores from two repetitions were merged and tested using an ordinary one-way ANOVA (**p ≤ 0.01). ns = non-significant.

The absence of disease development in Res | Sus plants can be caused by; 1) *Fol007* proliferation being contained in the resistant rootstock, preventing colonization of the susceptible scion, or 2) *Fol007* colonizes susceptible scions, but a mobile compound or signal from the resistant rootstock suppresses its ability to cause disease. To distinguish between these options the presence of *Fol007* in the scion was examined. Stem pieces were excised at various plant levels (below and above the grafting point, at the cotyledon, and first true leaf node) and cultured on agar plates to monitor fungal outgrowth (**Figure 2**). *Fol* presence was confirmed above the grafting point in all grafting combinations in two out of three repetitions (**Figure 2B**). The amount of *Fol* outgrowth from stem slices collected below and above the grafting point was quantified from two independent repetitions (**Figures 2C, D**). A significantly lower amount of fungal growth was observed from stem slices collected from below the grafting point of Res | Sus stems compared to Sus | Sus, whereas Sus | Res showed a similar trend as Sus | Sus (**Figure 2D**). For stem slices collected from Res | Res, there appears to be less fungal outgrowth in comparison to Sus | Sus, although this difference is not statistically significant. There were no differences observed in the fungal outgrowth from stem slices collected above the grafting point. The pathogen reached the first true leaf node of all Sus | Sus and Sus | Res grafts in every repetition (**Figure 2B**). The stem colonization of Res | Res and Res | Sus grafts were very similar to each other, with 30-40%, 70-80%, and 75% being colonized till the first true leaf node in the first, second, and third repetition, respectively. Combining fungal stem colonization data from three repetitions revealed significant differences between Sus | Sus and Res | Res or Res | Sus, while no significant difference between Sus | Sus and Res | Sus. These data show that *I-2* rootstocks do not prevent the colonization of susceptible scions by *Fol* but do reduce the colonization and prevent disease development. As the fungus is contained in the vessels of the susceptible scion we hypothesized that resistant roots signal immune responses to the shoot that may involve translocation of specific antifungal compounds via the xylem to restrict *Fol* proliferation and pathogenicity.

### Diverse levels of *Fol* secretory proteins in xylem sap of *I-1*, *I-2*, *I-3*, and *I-7* resistant lines

**Figure 3:**
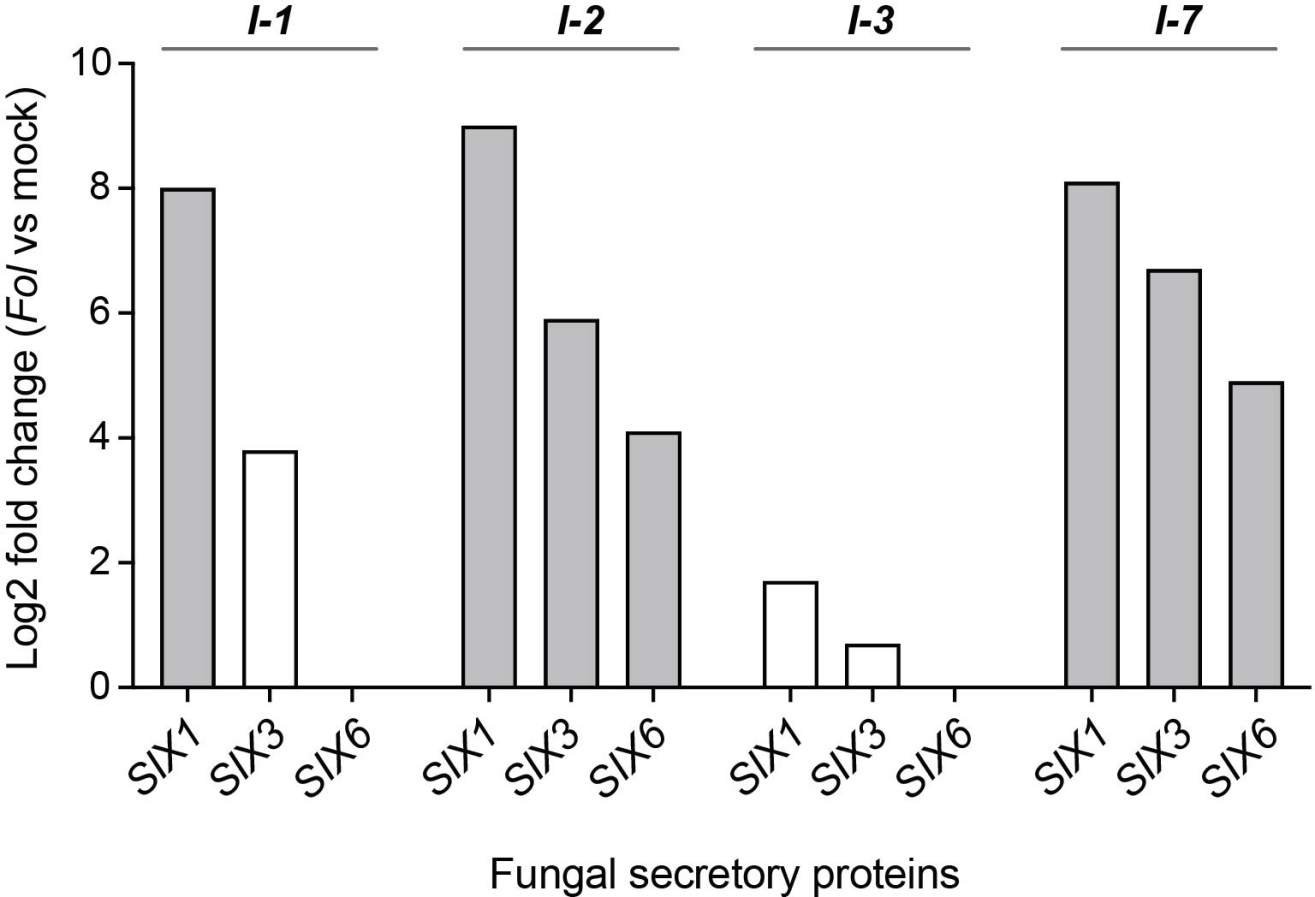
Fungal SIX proteins are present in xylem sap of resistant *I-1*, *I-2*, *I-3*, and *I-7* lines inoculated with *Fol*. Log2 fold change values indicate relative abundance differences of fungal secretory proteins SIX1 (Avr3), SIX3 (Avr2), and SIX6 between *Fol*- and mock-inoculated resistant lines (four or five repetitions). Grey bars represent differentially accumulating SIX proteins based on a Student’s T-test.

Avirulent *Fol* strains colonize the stems of resistant plants, and in *I-2* plants *Fol* has been shown to colonize the xylem vessels (Mes et al., 2000; van der Does et al., 2019). To test whether the absence of disease symptom development is due to a lack of effector secretion by the fungus, the xylem sap collected from *I-1*, *I-2*, *I-3*, and *I-7* lines infected with avirulent *Fol* was examined for the presence of *Fol* proteins (**Supplementary Table 1**). For xylem sap collection, four-week-old tomato plants were mock- and *Fol*-inoculated with the corresponding avirulent *Fol* race. Concentrated xylem sap samples were subjected to LC-MS analysis to quantify and identify fungal proteins. Secreted Into Xylem (SIX) proteins were found in all samples and were most abundant in the xylem sap collected from *I-1*, *I-2*, and *I-7* lines. SIX1, also known as Avr3 (A0A0C4DI36) was identified as a Differentially Accumulating Protein (DAP) in *I-1*, *I-2*, and *I-7* when compared to mock-inoculated plants (**Figure 3**). SIX3, or Avr2 (A0A0C4DI32), and SIX6 (A0A0C4DHX7) were identified as DAPs in *I-2* and *I-7* plants. SIX1 and SIX3 were also detected in I-3 xylem sap samples but only in one out of the five repetitions. These findings corroborate the presence of the fungus in the vasculature of resistant plants and show that it secretes effector proteins, albeit at varying abundances.

The difference in the SIX protein amounts in the sap likely reflects the amount of fungal biomass and/or the extent of stem colonization. Previous studies already reported different degrees of stem colonization of *I-1*, *I-2*, and *I-3* plants by avirulent *Fol* strains (van der Does et al., 2019). However, it remains unknown whether in *I-7* lines *Fol* race 3 (Fol029) can reach and colonize the stem, and if so, to what degree. To address this, ten-day-old resistant (MM+*I-7*) and susceptible (C32) tomato seedlings were mock- and *Fol029*-inoculated. At nine days post inoculation (dpi), stem fragments were harvested and fungal presence was assessed. *I-7* plants were colonized by *Fol* up to 63% of their height, while susceptible plants were colonized almost fully (96%) (**Supplementary Figure 1**). These findings confirm the presence of *Fol* in the xylem system, secreting SIX proteins, albeit with varying abundance across resistance lines.

### I-1, I-2, I-3, and I-7 R-protein activation results in the accumulation of a set of unique and shared proteins in the xylem sap

**Figure 4:**
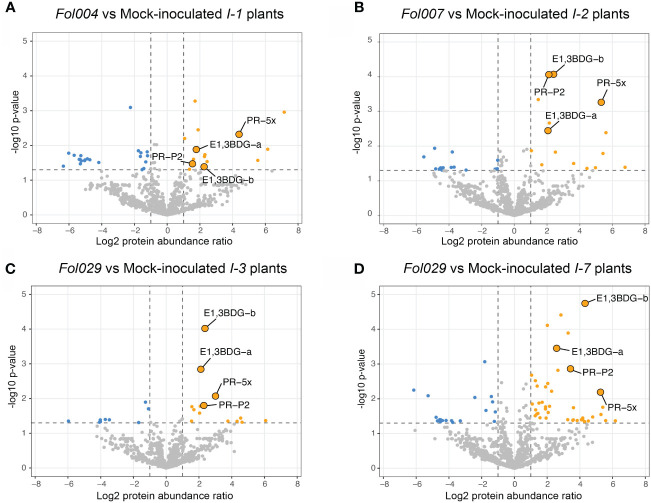
*I-1*, *I-2*, *I-3*, and *I-7 R*-gene mediated resistance effects on tomato xylem sap proteome. Volcano plots represent -log10 p-values and log2 protein abundance ratios by comparing mock- and *Fol*- inoculated resistant lines (four or five repetitions). **(A)** Volcano plots compare mock- and *Fol004*- inoculated *I-1* resistant line, **(B)** mock- and *Fol007*- inoculated *I-2* resistant lines, **(C)** mock- and *Fol029*- inoculated *I-3* resistant lines, and **(D)** mock- and *Fol029*- inoculated *I-7* resistant lines. Grey dots represent non-differentially accumulating proteins (DAP), blue dots are downregulated DAPs (-log10 p-value > 1.3, log2 protein abundance ratio < -1), and orange dots are upregulated DAPs (-log10 p-value > 1.3, log2 protein abundance ratio >1). Four upregulated DAPs have been identified in all resistant lines and are highlighted in each volcano plot: PR-5x, PR-P2, and two glucan endo-1,3-β-D-glucosidase (endo.3BDG-a/b).

To identify proteins associated with *Fol* resistance, quantitative proteome analysis was conducted on the xylem sap of the resistant lines following mock- or *Fol*-inoculation. The availability of four structurally distinct R-receptors (I-1, I-2, I-3, or I-7) within a single pathosystem provides the opportunity to identify unique and shared proteins that differentially accumulate upon *Fol* inoculation. LC-MS analysis of concentrated xylem sap revealed on average 860 plant proteins per sample (**Supplementary Table 1**). A minority of the identified proteins were classified as DAPs, as depicted in the volcano plots: 4.7% in I-1, 3.3% in I-2, 2.8% in I-3, and 7% in I-7 (**Figures 4A-D**).

**Figure 5:**
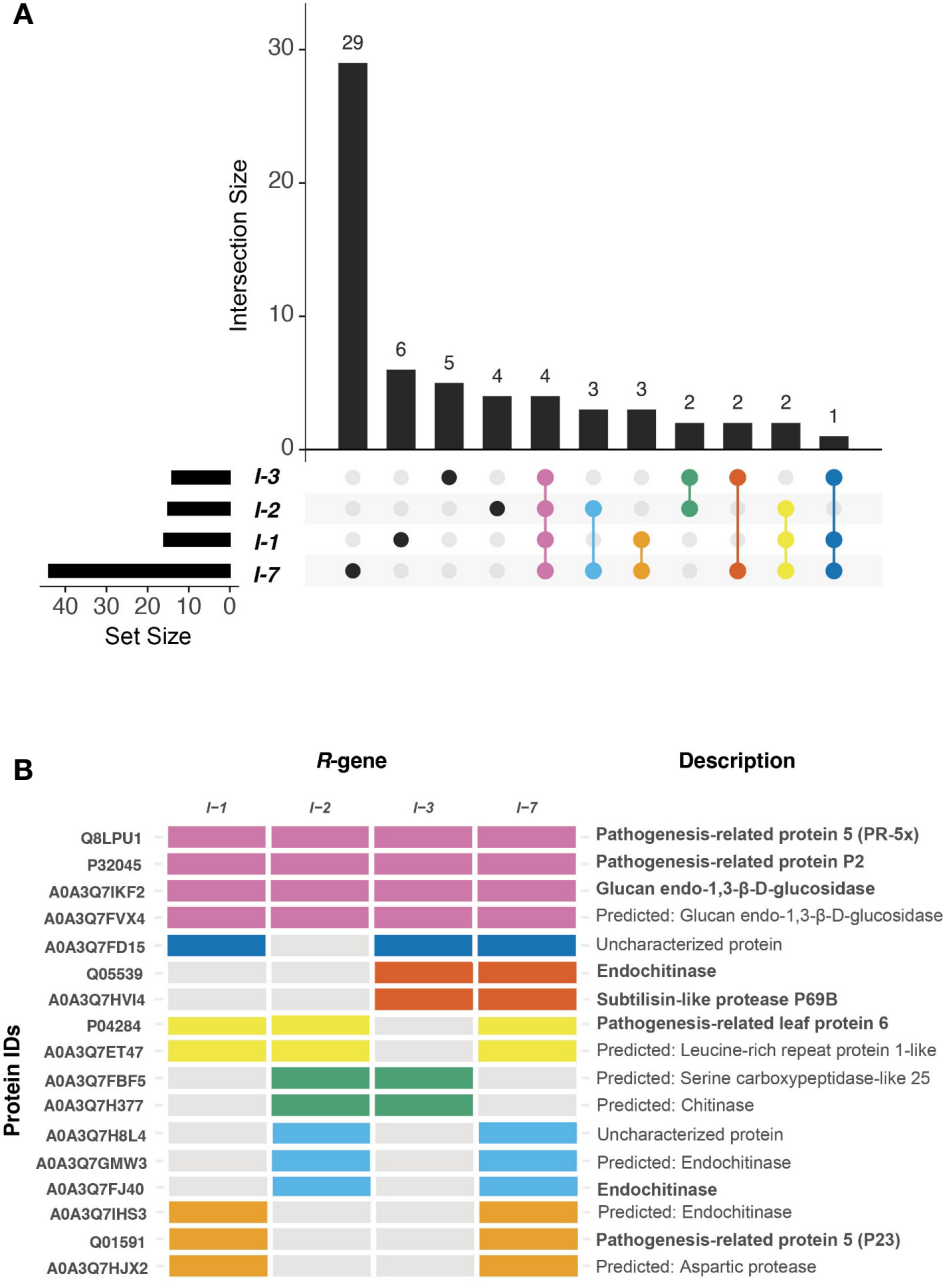
*I-1*, *I-2*, *I-3*, and *I-7* induce the accumulation of unique and shared DAPs. **(A)** UpSet plot compares proteins differentially upregulated during *R*-gene-mediated resistances. The horizontal bar graph indicates the total number of DAPs per resistant line. The intersection bar graph displays the number of DAPs that are unique (black dot) or shared (colored dot) between resistant lines. **(B)** The tile plot shows the presence-absence of DAPs that are present in at least two *R*-gene lines. The tile plot color corresponds to the Upset plot dots.

To identify shared and unique responses induced by the different R-proteins to *Fol*, the upregulated proteins in the DAP list were compared using an UpSet plot (**Figure 5A**). These comparative analyses revealed a unique and shared set of upregulated DAPs among *I-1*, *I-2*, *I-3*, and *I-7*. All four *Fol*-inoculated *I*-gene lines exhibited differential accumulation of four proteins: PR-5x (Q8LPU1), PR-P2 (P32045), and two glucan endo-1,3-β-D-glucosidases (A0A3Q7IKF2, A0A3Q7FVX4) (**Figure 5B**). The p-value and protein abundance ratio of these four DAPs vary between the resistant lines (**Figures 4A-D**). Apart from this common set of pathogenesis-related proteins, subsets were observed that were shared between two or three resistant lines. For example, *Fol*-inoculated *I-1* and *I-7* lines show differential accumulation of an endochitinase (A0A3Q7IHS3), PR-P23 (Q01591) and an aspartic protease (A0A3Q7HJX2); *I-2* and *I-3* both accumulate a serine carboxypeptidase-like protein (A0A3Q7FBF5) and a chitinase (A0A3Q7H377); *I-2* and *I-7* share an uncharacterized protein (A0A3Q7H8L4) and two endochitinases (A0A3Q7GMW3, A0A3Q7FJ40); *I-3* and *I-7* an endochitinase (Q05539) and a subtilisin-like protease, P69B (A0A3Q7HVI4); *I-1*, *I-2*, and *I-7* a PR-6 (P04284) and a leucine-rich repeat-like protein (A0A3Q7ET47); and *I-1*, *I-3* and *I-7* an uncharacterized protein (A0A3Q7FD15). In addition, DAPS unique to a single resistant line were identified (**Table 1**). For instance; *I-1* plants showed differential accumulation of two chitin-degrading enzymes, a peroxidase, and a xylose synthase; *I-2* a glycoprotein, a redox protein, and a rapid alkalization factor; *I-3* a pathogenesis-related 5 protein, a sugar-degrading enzyme, and a protease; *I-7* two pathogenesis-related protein 1 isoforms, tobacco stress-induced protein 1, four complex carbohydrate degrading enzymes, and three proteases. The comparative analysis suggests that in addition to distinct immune responses against *Fol*, *I-1*, *I-2*, *I-3*, and *I-7* lines accumulate a common set of four PR proteins as DAPs in the xylem sap of inoculated plants.

**Table 1:** Each *I*-mediated resistance (*I-1*, *I-2*, *I-3*, and *I-7*) induces accumulation of a unique set DAPs.

*R*-gene line	Protein ID	Description
*I-1*	A0A3Q7E9J6	UDP-D-xylose synthase 2
	A0A3Q7IIQ3	Endochitinase 3-like
	A0A3Q7IN81	Enolase
	Q05538	Basic 30 kDa endochitinase
	A0A3Q7H0G7	Dirigent protein 25 isoform X2
	A0A3Q7F8E3	Peroxidase 63
*I-2*	A0A3Q7EN81	Rapid alkalinization factor
	A0A3Q7G526	Blue copper protein
	A0A3Q7IS69	GDP-dissociation inhibitor 1
	A0A3Q7HEE3	Epidermis specific secreted glycoprotein EP1
*I-3*	A0A3Q7F6T3	Pathogenesis related protein 5
	A0A3Q7IIS3	Uncharacterized protein
	A0A3Q7EN73	Uncharacterized protein
	A0A3Q7GBV0	Endoglucanase 2
	A0A3Q7IPI3	ATP-dependent Clp protease
*I-7*	A0A3Q7FE00	Glutaminyl-peptide cyclotransferase
	Q08697	Pathogenesis-related protein 1 A1
	A0A3Q7HXW1	Pathogenesis-related protein 1 A2
	A0A3Q7HVR3	Uncharacterized protein
	A0A3Q7HKU9	Aspartic proteinase Asp1
	A0A3Q7J2Z7	Polygalacturonase
	A0A3Q7I7U0	Stem-specific protein TSJT1
	A0A3Q7HIV8	Cellulose synthase-like protein E1
	A0A3Q7GSL2	Superoxide dismutase
	A0A3Q7F3P0	Epidermis specific secreted glycoprotein EP1-like
	A0A3Q7JPB1	Glucan endo-1,3-beta-glucosidase 14
	A0A3Q7HS86	GSDL-like lipase/acylhydrolase
	A0A3Q7I9H4	Tobacco stress-induced 1
	A0A3Q7IGR5	Receptor-like protein 33
	A0A3Q7EEZ8	Glucan endo-1,3-beta-glucosidase B
	A0A3Q7EP77	Polygalacturonase
	A0A3Q7JEN8	Dihydrolipoyl dehydrogenase
	A0A3Q7EHR7	Basic 7S globulin
	A0A3Q7HJU0	Pectin acetylesterase 8
	A0A3Q7ESD4	Inactive leucine-rich repeat receptor kinase XIAO
	A0A3Q7HTM2	Subtilisin-like protease P69G
	A0A3Q7ESI1	Phospholipase C2
	A0A3Q7J0R2	Ubiquitin carboxyl-terminal hydrolase 12-like isoform
	P85997	Kunitz trypsin inhibitor 2-like
	K4C9C4	CLAVATA3/ESR (CLE)-related protein 9
	O48625	Miraculin
	A0A3Q7EU87	Trisosephosphate isomerase chloroplastic
	A0A3Q7FPY9	Cysteine protease
	A0A3Q7F218	Alpha-glucosidase-like

## Discussion

In this study, we demonstrate the effectiveness of an *I-2*-resistant rootstock to protect a susceptible scion from developing Fusarium wilt symptoms, notwithstanding that *Fol* colonizes the scion tissues and secretes effector proteins. The containment of *Fol* in the susceptible tissue suggests a potential role of xylem sap in Fusarium resistance, by transporting virulence-suppressing components from the root into the shoot. Comparative proteomics analyses revealed distinct PR protein profiles in xylem sap obtained from *Fol*-inoculated resistant lines (*I-1*, *I-2*, *I-3*, and *I-7*). Notably, despite their structural diversity, all four immune receptors triggered the differential accumulation of a common set of just four PR proteins (PR-5x, PR-P2,1, and two glucan endo-1,3-β-D-glucosidases). These proteins are, therefore, prime candidates for being involved in restricting fungal proliferation in the foliage, thereby preventing disease development. Interestingly, the extent of fungal colonization in the resistant plants, as monitored by both the height of host colonization, the amount of fungal outgrowth, and the accumulation of SIX1, SIX3, and SIX6 proteins, differed depending on the *R*-gene present. *I-3* lines displayed the strongest reduction of fungal colonization, *I-1* exerted somewhat weaker responses, while *I-2* and *I-7* showed the poorest containment, corresponding to the highest SIX protein abundance.

Our analyses revealed differential accumulations of PR-5x in all resistant lines (*I-1*, *I-2*, *I-3*, and *I-7*) at one wpi. PR-5x was identified previously in the xylem sap of susceptible plants inoculated with a virulent *Fol* race 1 at one, two, and three wpi (Rep et al., 2002). Furthermore, PR-5x was identified as the sole DAP in the xylem sap of resistant *I-2-*lines at two wpi, showing a 158-fold increase in accumulation. Inoculation of susceptible plants resulted in a 17-fold increase in PR-5x abundance (de Lamo et al., 2018). Apart from its putative involvement in resistance to *F. oxysporum*, PR-5x was also identified as a DAP in xylem sap from tomato plants resistant to the bacterial vascular pathogen *R. solanacearum* (Planas-Marquès, 2020). Additionally, two other PR-5 isoforms were identified in our study: PR-P23 (Q01591) in *I-1* and *I-*7 lines and another PR-5 isoform (A0A3Q7F6T3) in *I-3* plants. Two PR-5 isoforms identified in the apoplast were reported to exhibit direct antifungal activity by disrupting the lipid bilayer of the pathogen (Vigers et al., 1992). Overexpression of specific PR-5 members enhanced resistance to *Phytophthora infestans*, *Rhizoctonia solani*, *Fusarium graminearum*, and *Alternaria alternata* in potato, rice, wheat, and tobacco, respectively (Liu et al., 1994; Chen et al., 1999; Datta et al., 1999; Anand et al., 2003). These studies suggest a potentially direct activity of PR-5 proteins in the xylem sap against vascular pathogens. Whether the xylem-localized PR-5 isoforms identified in this study have direct antifungal activity awaits additional studies.

Besides PR-5x, differential accumulation of PR-P2 and two endo-β-glucosidases was observed in the *I-1*, *I-2*, *I-3*, and *I-7* resistant lines. These PR proteins were previously not detected as DAPs in the xylem sap of *Fol*-inoculated resistant *I-2* plants (de Lamo et al., 2018). This discrepancy could be attributed to different sampling time points for xylem sap collection (two wpi versus one wpi in this study) and/or increased sensitivity of the protein purification method and LC-MS machine used. In this study, ~ 700 plant proteins were identified in mock-inoculated *I-2* plants at 1wpi, while the previous study revealed ~ 285 plant proteins at 2wpi using the same plant line and inoculation procedure (de Lamo et al., 2018). PR-P2 belongs to the PR-4 protein subgroup II, due to the absence of a chitin-binding domain. Tomato lines that exhibit resistance to *Cladosporium fulvum* show a faster accumulation of PR-P2 as compared to susceptible lines (Linthorst et al., 1991). A wheat PR-4 hydrolyses *Fusarium culmorum* RNA and inhibits fungal growth (Guevara-Morato et al., 2010). β-glucosidases hydrolyze glucose polymers such as cellulose (Bhat and Bhat, 1997). Overexpression in maize has been associated with enhanced resistance against both the oomycete pathogen *P. aphanidermatum* and the Asian corn borer *Ostrinia furnacalis* (Liu et al., 2023). As the latter does not have glucose polymers in its exoskeleton the action of the protein might be to release DAMPs amplifying the immune response. To explore a potential causal relationship between Fusarium resistance and PR-5x, PR-P2, and the two-glucan endo-1,3-β-D-glucosidases future experiments could focus on generating overexpression and knock-out tomato lines to test these genes either individually or in combination, and by testing purified proteins for direct antifungal activity.

Our comparative proteomics study also unveiled diverse PR proteins that exhibited differential accumulation in the xylem sap of distinct resistant lines, namely PR-1, PR-2, PR-3, PR-6, and PR-7. PR-1 (Q08697; A0A3Q7HXW1) undergoes proteolytic cleavage to release the CAPE1 peptide that is involved in plant immune responses (Chen et al., 2014). Overexpression of *PR-1* inhibited oomycete and rust fungus colonization in tomato and bean plants, respectively (Rauscher et al., 1999; Li et al., 2011). Co-silencing of PR-1 and PR-5, which were reported to interact, enhanced susceptibility to leaf rust in wheat plants (Wang et al., 2020). PR-2, β-1,3-glucanases (A0A3Q7GBV0), inhibit fungal growth by hydrolyzing β-1,3-glucans, a major structural component of fungal cells (Mauch et al., 1988; Sela-Buurlage et al., 1993; Balasubramanian et al., 2012). Co-expression of β-1,3-glucanases and chitinases in transgenic tomato plants resulted in enhanced *Fol* resistance (Jongedijk et al., 1995). PR-3, chitinases (A0A3Q7IHS3; A0A3Q7H377; A0A3Q7GMW3, A0A3Q7FJ40, Q05539) hydrolyze chitin, a major fungal cell wall component, thereby also indirectly activating defense mechanisms by releasing PAMPs (Ohnuma et al., 2012). Wheat chitinase overexpression in tomato plants enhanced *Fol* resistance (Girhepuje and Shinde, 2011). PR-7s, P69B (A0A3Q7HVI4), and P69G (A0A3Q7HTM2) belong to the family of subtilisin-like proteases, and these show differential accumulation in the xylem sap of *R. solanacearum* resistant tomato plants (Planas-Marquès, 2020). Transient expression of *P69B* and *P69G* in *N. benthamiana* plants reduced *R. solanacearum* proliferation (Zhang et al., 2023). PR-6 (P04284), a proteinase inhibitor, blocks pathogen protease activity, reducing its proliferation (Lopes et al., 2009; Rodríguez-Sifuentes et al., 2020). Each *R*-gene induces the accumulation of a unique subset of these PR proteins. This difference might be attributed to the diverse structures and cellular localization of the R-receptors activating distinct and partially overlapping signaling pathways. Despite these differences, the accumulation of a shared set of PR proteins in the xylem sap, many of which also accumulate in apoplastic spaces upon immune activation, indicates a common defense response (Joosten and de Wit, 1989; van Loon et al., 2006). The role of the described PR proteins in vascular resistance could be studied using disease assays on tomato plants in which these genes are overexpressed or knocked-out. Possibly these assays can be performed on hairy roots generated after transformation with *Rhizobium rhizogenes* to accelerate the generation of stably transformed roots. Besides *in planta* assays, direct antifungal activity of the proteins could be assessed using purified recombinant protein and *in vitro* antifungal assays.

Plants protected by *I-1-*, *I-2-*, and *I-3-*mediated resistance allowed *Fol* to colonize stems up to 41%, 67%, and 29% of their total height, respectively (van der Does et al., 2019). We show that *I-7-*mediated resistance permits *Fol* to colonize up to 63% of the plant stem height, while susceptible plants were almost completely colonized (96%). Varying levels of several *Fol* SIX proteins were detected in the xylem sap of the resistant *I-1*, *I-2*, *I-3*, and *I-7* lines at 1wpi. It is unknown whether there are differences in SIX secretion efficiencies among the three *Fol* races (*Fol004*, *Fol007*, and *Fol029*) used, nonetheless, we observe a strong correlation between the extent of fungal stem colonization and the abundance of SIX proteins in the xylem sap. Notably, *I-3* exhibited the lowest stem colonization and the lowest abundance of SIX proteins, while *I-2* and *I-7* showed the highest stem colonization along with the highest SIX protein quantity. Possibly, the ETI responses triggered by the distinct *I* genes affect effector secretion and/or activity to different extents resulting in differences in host colonization by the fungus. Alternatively, the differences in *Fol* proliferation among the resistant lines may relate to differences in the expression patterns of the *R*-genes in the root tissues (**Supplementary Figure 2**). *I-1* is expressed in the epidermis and lateral root cap*; I-3* in the exodermis, two cortex cell layers, and endodermis; *I-7* in the xylem and epidermis maturation zone (Kajala et al., 2021). Based on an *I-2* promotor GUS reporter system high expression of *I-2* was observed in xylem parenchyma cells (Mes et al., 2000). These data suggest that *R*-gene activation in multiple outer root tissue layers may result in reduced *Fol* colonization and abundance in the vasculature of the stem. On the contrary, when *Fol* is recognized in the xylem parenchyma cells, there is an increase in *Fol* colonization and abundance. It is tempting to speculate that earlier detection allows the plant to mount a faster and more effective defense response. This early response may include the accumulation of PR proteins in the xylem (Linthorst et al., 1991) and/or the development of mechanical barriers. Fortification of the vasculature occurs during *Fusarium oxysporum* f. sp. *pisi* and *R. solanacearum* colonization of pea and tomato plants, respectively (Bani et al., 2018; Kashyap et al., 2022). To investigate whether the timing of the response is a crucial factor, xylem sap could be collected at earlier time points. A comparison could then be made between an *R*-gene expressed in the outer root tissue layer and one expressed in xylem parenchyma cells. To establish a stronger link between expression patterns and *Fol* resistance, and to rule out any influence from structural differences between *R*-genes, the promoter regions of different *R*-genes could be swapped and tested for *Fol* colonization potential.

## Data availability statement

The datasets presented in this study can be found in online repositories. The names of the repository/repositories and accession number(s) can be found in the article/**Supplementary Material** and the Mass spectrometry data are available in the MassIVE repository (https://massive.ucsd.edu/) via the dataset identifier PXD051503.

## Author contributions

MŠ: Conceptualization, Data curation, Formal analysis, Funding acquisition, Investigation, Methodology, Resources, Validation, Visualization, Writing – original draft, Writing – review & editing. GK: Data curation, Formal analysis, Investigation, Resources, Software, Visualization, Writing – review & editing, Methodology. MR: Conceptualization, Funding acquisition, Supervision, Writing – review & editing. FT: Conceptualization, Data curation, Formal analysis, Funding acquisition, Investigation, Methodology, Project administration, Resources, Supervision, Validation, Visualization, Writing – original draft, Writing – review & editing.

## Supplementary material

Supplementary Figure 1 *Fol029* (race 3) can colonize *I-7* tomato plants. Ten-day-old *Fol029* susceptible (cv. C32) or *I-7* resistant (cv. MM+*I-7*) tomato seedlings were mock- or *Fol*- inoculated. **(A)** Nine dpi, the distance between the soil and the tip of the stem was measured. The whole stem was cut into approx. 8mm stem sections and placed on agar plates in a consecutive order. **(B)** Representative scans of three days old plates display *Fol* outgrowth from stem sections. **(C)** Each bar graph illustrates one repetition, which shows the average fungal colonization height of ten susceptible or resistant plants in cm. Each blue bar represents the average total stem height per treatment. The red bar represents the average height until where *Fol* was able to colonize the stem. Error bars indicate SD.

Supplementary Figure 2 *I-1*, *I-2*, *I-3*, and *I-7* spatial expression patterns in tomato roots. *I-1* (*Solyc11g011180*), *I-3* (Solyc11g055640), and *I-7* (Solyc08g077740) expression patterns are based on https://bar.utoronto.ca/eplant_tomato/and *I-2* (Solyc11g071430) on GUS reporter lines. Tissue layers with expression are colored in red (*I-1*, *I-3*, *I-7*) or orange (*I-2*).

Supplementary Table 1 Data annotation and statistical analyses of xylem sap proteomics data from mock or *Fol*-inoculated *I-1*, *I-2*, *I-3*, and *I-7* resistant lines. Every protein ID is either labeled as “Present” (identified in 4 of 5 or for *I-1* 3 of 4 repetitions) or “Marginal” (identified only in 1 of 5 or for *I-1* 1 of 4 repetitions). The table includes protein IDs, relative abundance values (LFQ), fasta headers, p-values, abundance ratio values, and peptide counts.
