# Supplementary figures and images for: Tomato R-gene-mediated resistance against Fusarium wilt originates in roots and extends to shoots via xylem to limit pathogen colonization
Source: Front Plant Sci. 2024 May 1;15:1384431. doi: 10.3389/fpls.2024.1384431 (PMC11094230; doi:10.3389/fpls.2024.1384431)

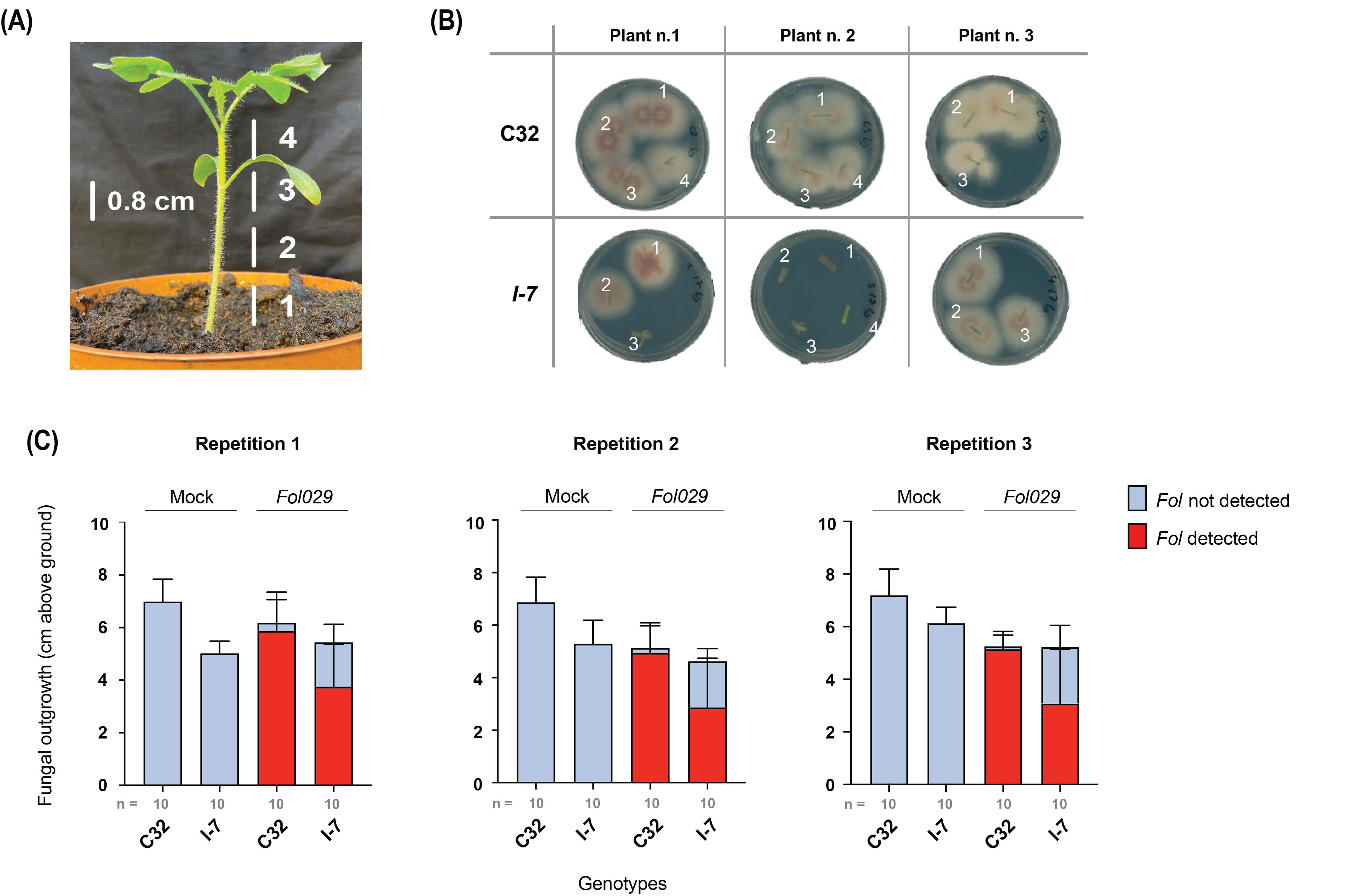

Supplement: Supplementary Figure 1 — Fol029 (race 3) can colonize I-7 tomato plants. Ten-day-old Fol029 susceptible (cv. C32) or I-7 resistant (cv. MM+I-7) tomato seedlings were mock- or Fol- inoculated. (A) Nine dpi, the distance between the soil and the tip of the stem was measured. The whole stem was cut into approx. 8mm stem sections and placed on agar plates in a consecutive order. (B) Representative scans of three days old plates display Fol outgrowth from stem sections. (C) Each bar graph illustrates one repetition, which shows the average fungal colonization height of ten susceptible or resistant plants in cm. Each blue bar represents the average total stem height per treatment. The red bar represents the average height until where Fol was able to colonize the stem. Error bars indicate SD. [file Image_1.jpeg]

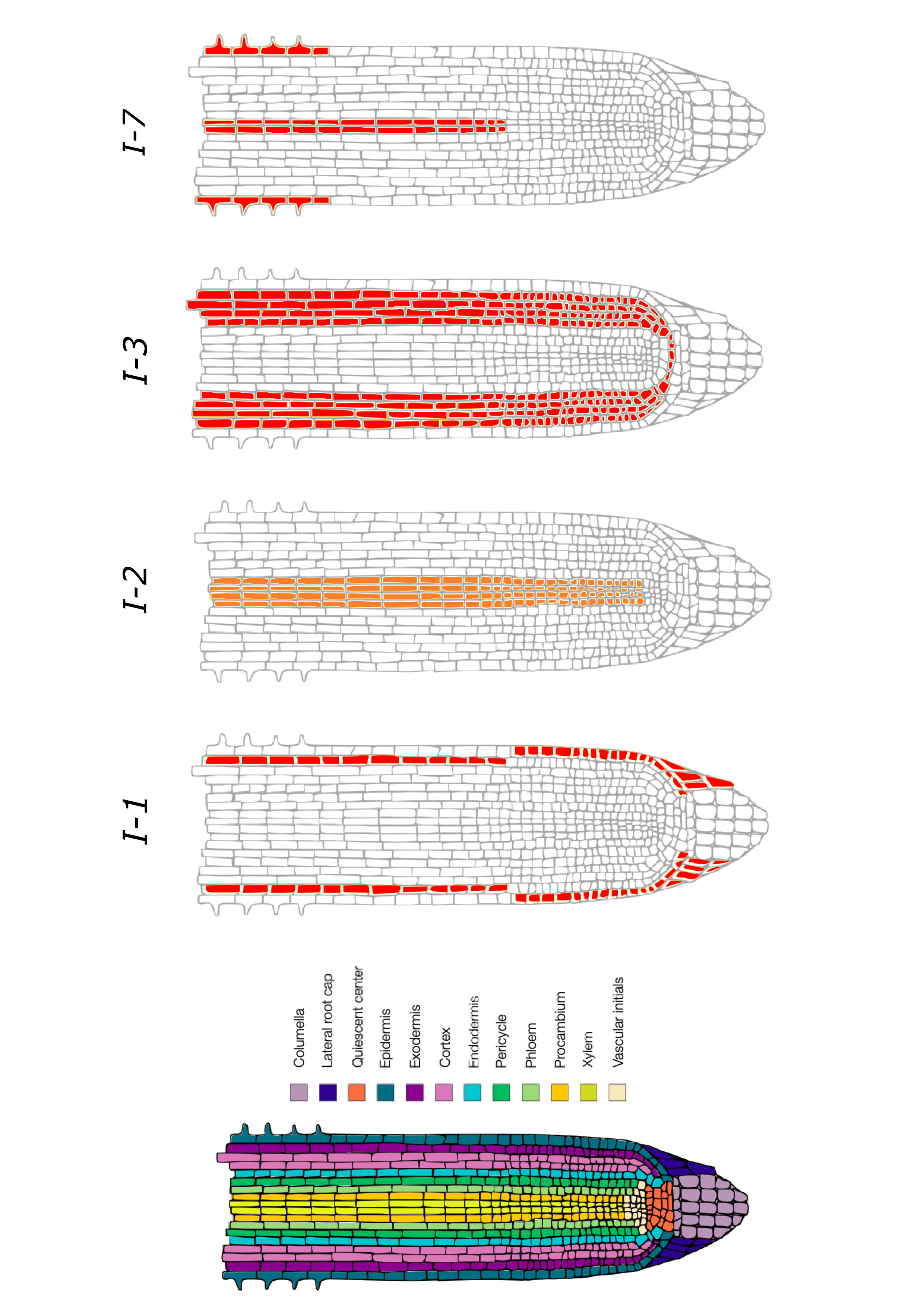

Supplement: Supplementary Figure 2 — I-1, I-2, I-3, and I-7 spatial expression patterns in tomato roots. I-1 (Solyc11g011180), I-3 (Solyc11g055640), and I-7 (Solyc08g077740) expression patterns are based on https://bar.utoronto.ca/eplant_tomato/and I-2 (Solyc11g071430) on GUS reporter lines. Tissue layers with expression are colored in red (I-1, I-3, I-7) or orange (I-2). [file Image_2.jpeg]
